# Supplementary material for: Virulent coliphages in 1-year-old children fecal samples are fewer, but more infectious than temperate coliphages
Source: Nat Commun. 2020 Jan 17;11:378. doi: 10.1038/s41467-019-14042-z (PMC6969025; doi:10.1038/s41467-019-14042-z)
Supplement: Supplementary file 1 — Supplementary Information [file 41467_2019_14042_MOESM1_ESM.pdf]

Virulent coliphages in 1-year-old children fecal samples are fewer, but more infectious than temperate coliphages

Mathieu et al.

Supplementary files

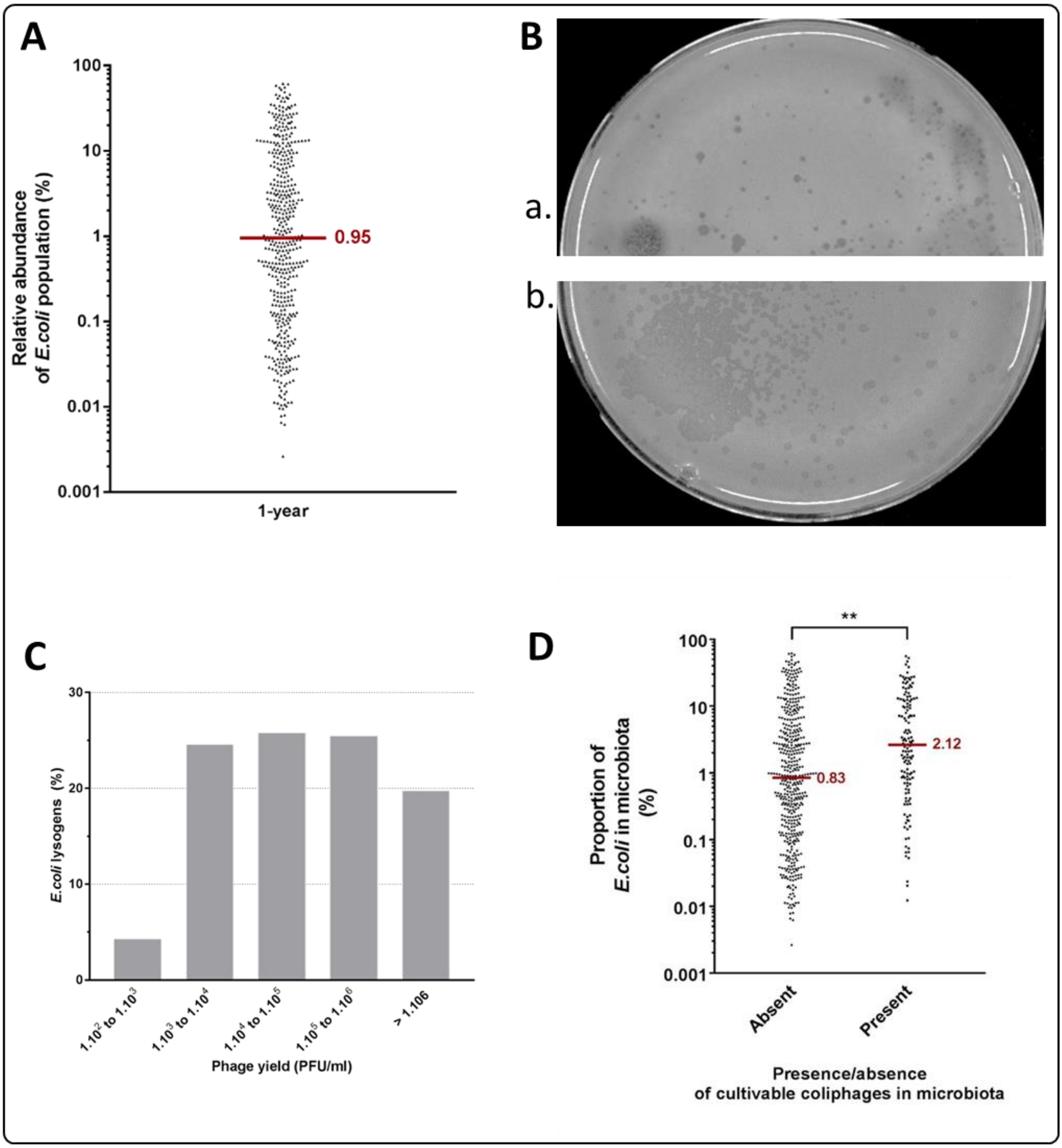

Supplementary Figure 1: *E. coli* populations in fecal samples of 1-year-old infants. A- Ratio of *E. coli* to total OTUs in fecal samples from 1-year-olds. The red line corresponds to the median value. **B-** Lysis plaque assays. The supernatant of overnight (O/N) cultures of two *E. coli* strains were filtered and 10 µl were spotted on plate before adding the soft agar containing *E. coli* MAC1403 strain. Lysis plaques were observed after an O/N incubation at 37°C. Two half plates with different supernatants are shown (a and b). **C-** Distribution of phage yields in *E. coli* lysogen supernatants (PFU/ml). **D-** The fraction of *E. coli* to total microbiota OTU is significantly higher among fecal samples from which cultivable coliphages were obtained. ** Mann-Whitney two-tailed test, P value < 0.0001. Source data are provided as a Source Data file.

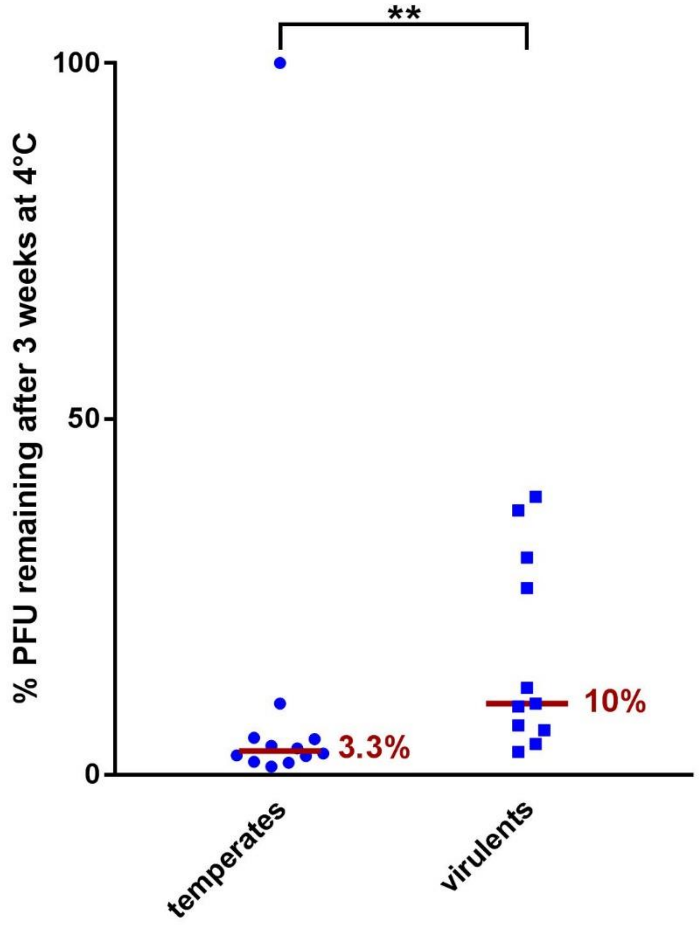

Supplementary Figure 2: Coliphage stability at 4°C. Lysates of 11 temperate and 12 virulent coliphages stored at 4°C were titrated at day 0 and 35 after phage production. Each point represents the average of the decay (% of initial titer) on two independent stocks. The difference in stability between temperate and virulent is significant (**, Mann-Whitney two-tailed test, P= 0.0059). Source data are provided as a Source Data file.

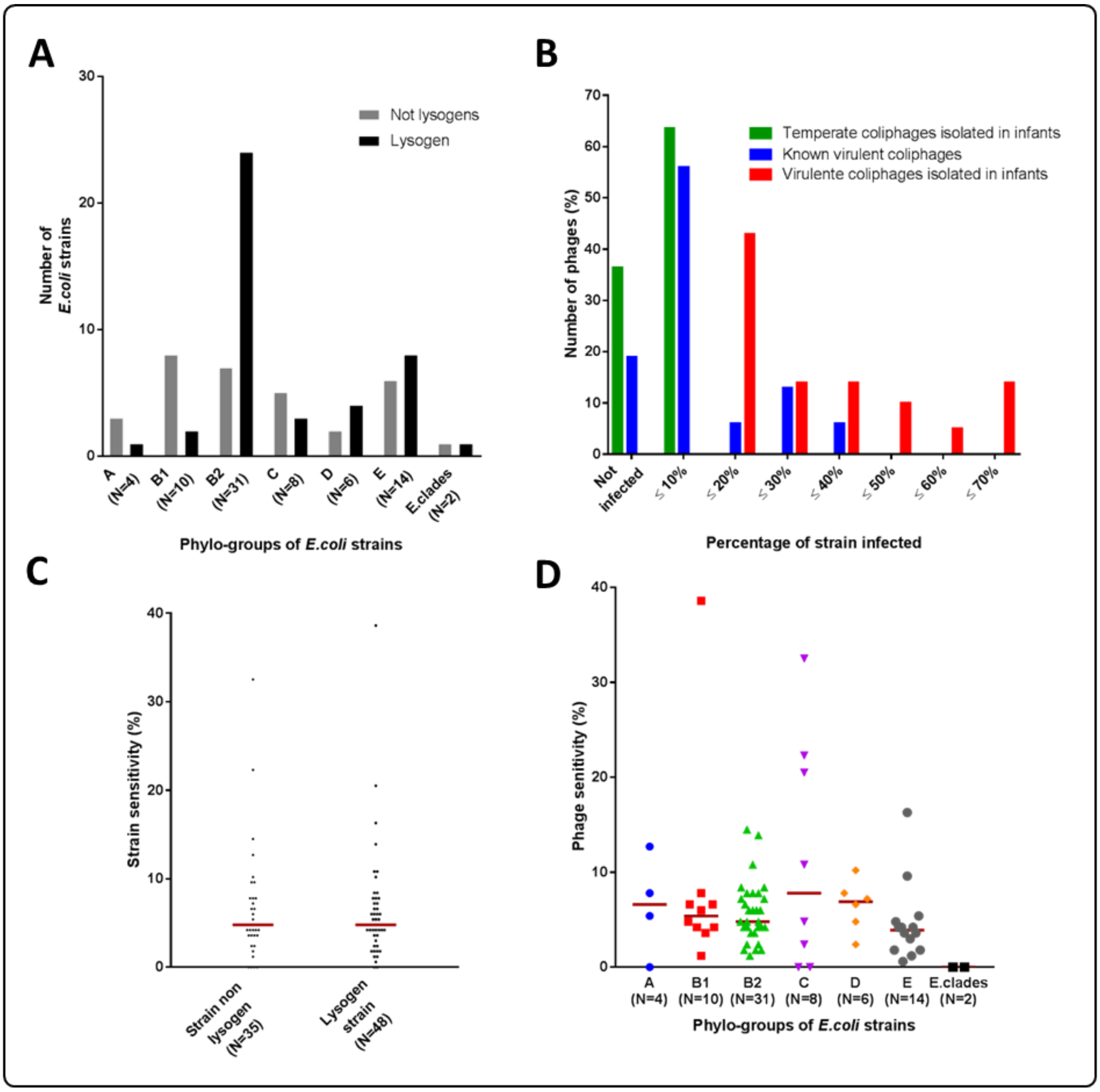

Supplementary Figure 3: Phage-host interactions and phylogroups. A- Number of *E. coli* lysogens as a function of strain phylogroup. B2 has significantly more *E. coli* lysogens than the average (74 %). A and B1 have significantly less. **B-** Three distinct profiles of coliphage infectivity on 75 natural *E. coli* strains according to the phage life-style (temperate or virulent). **C-** Phage-sensitivity of the 75 *E. coli* strains according to their lysogenic character. **D-** Phage sensitivity of *E. coli* strains stratified by phylogroup. Results are not significantly different between phylo-groups. Red lines: median values. Source data are provided as a Source Data file.

Suppl. figure 4A

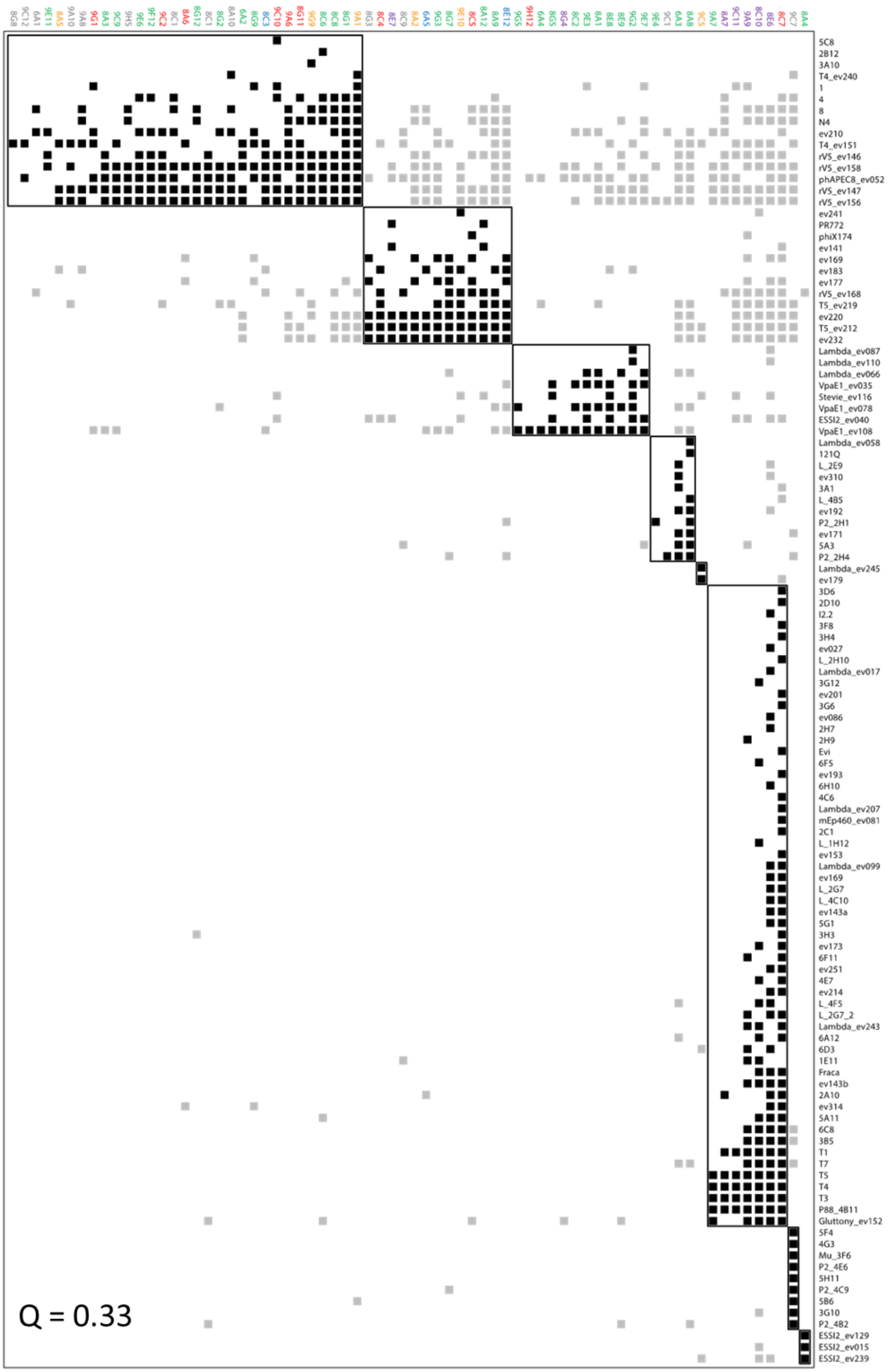

Suppl. figure 4B

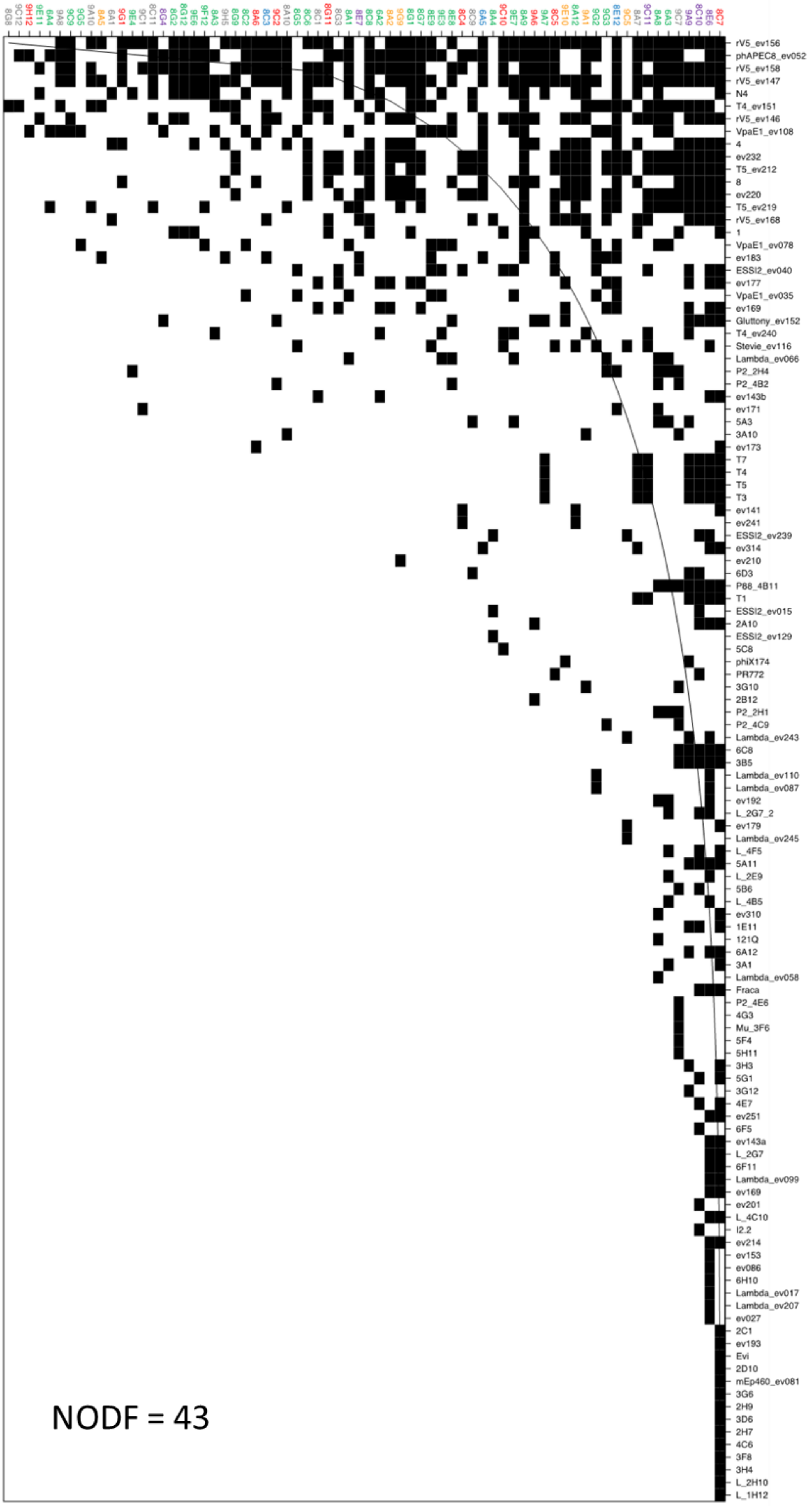

Supplementary Figure 4: **A- Modular arrangement of the interaction matrix.** The matrix is composed of 70 *E. coli* isolates and 116 phages. Bacterial isolates are shown on the x axis and colored according to their phylogroup (see Table 1) and phages are shown on the y axis. Each square (grey or black) indicates an interaction between a phage and an isolate, *i.e.* the isolate was sensitive to this phage. Each rectangle filled with black squares represents a module. The modularity level (Q), estimated with the lpbrim package in R, is indicated in the lower left corner**. B- Nested arrangement of the interaction matrix.** Same coding as in A, the matrix is now arranged to maximize nestedness. The black curve is an isocline and represents a perfect nestedness for a matrix with this dimension. The nestedness, estimated with the NODF function (vegan package in R), is indicated in the lower left corner.

Suppl. figure 5

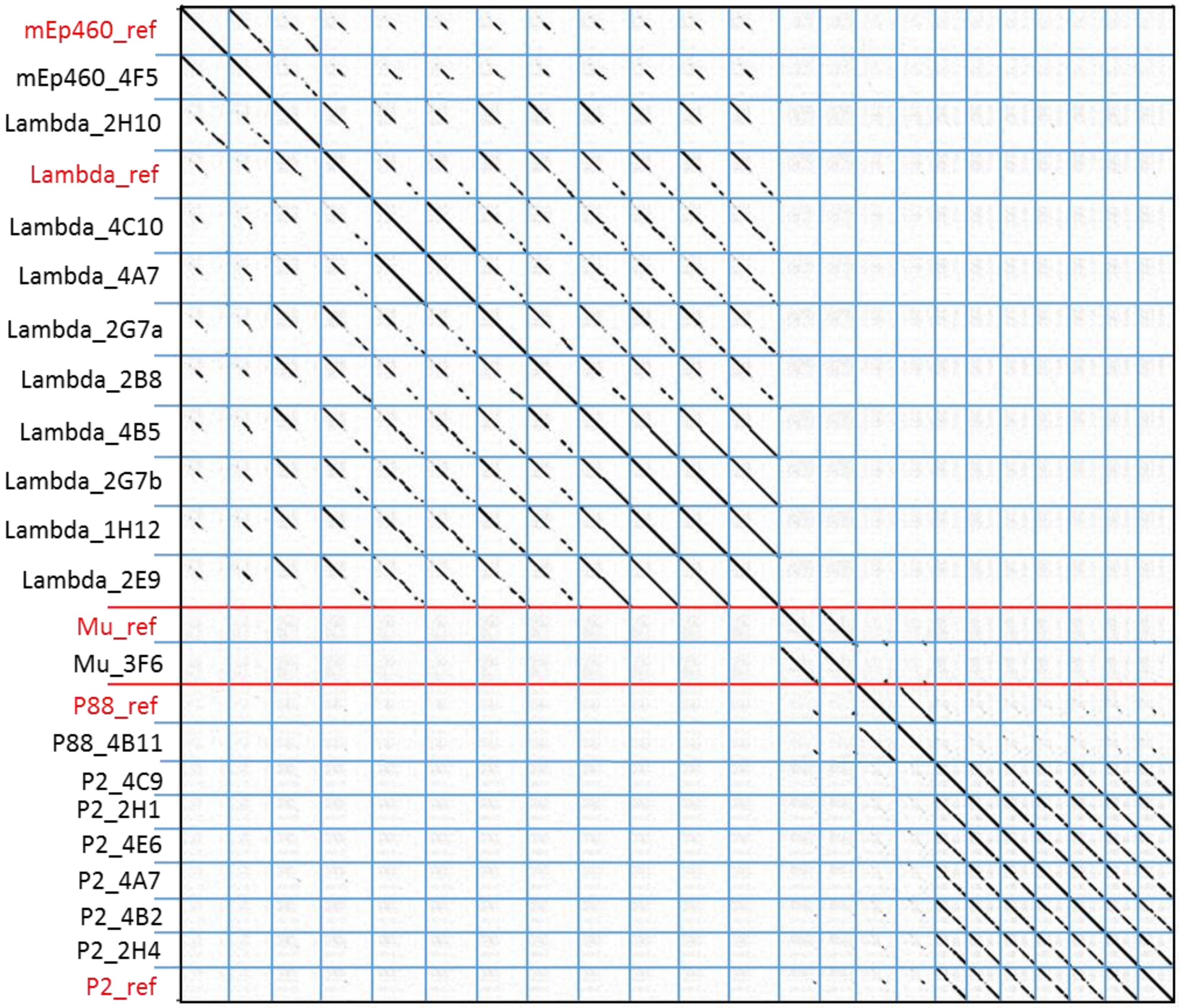

Supplementary Figure 5: Dot plot analysis of 18 coliphage complete genomes produced by natural strains isolated from 1 year-old children fecal samples. Genomes were aligned with reference sequences and compared with a Gepard[67] word size of 10. Red lines separate phage clusters and blue lines separate genomes within the clusters. Temperate phages are in black. Reference phages are in red.

Suppl. figure 6

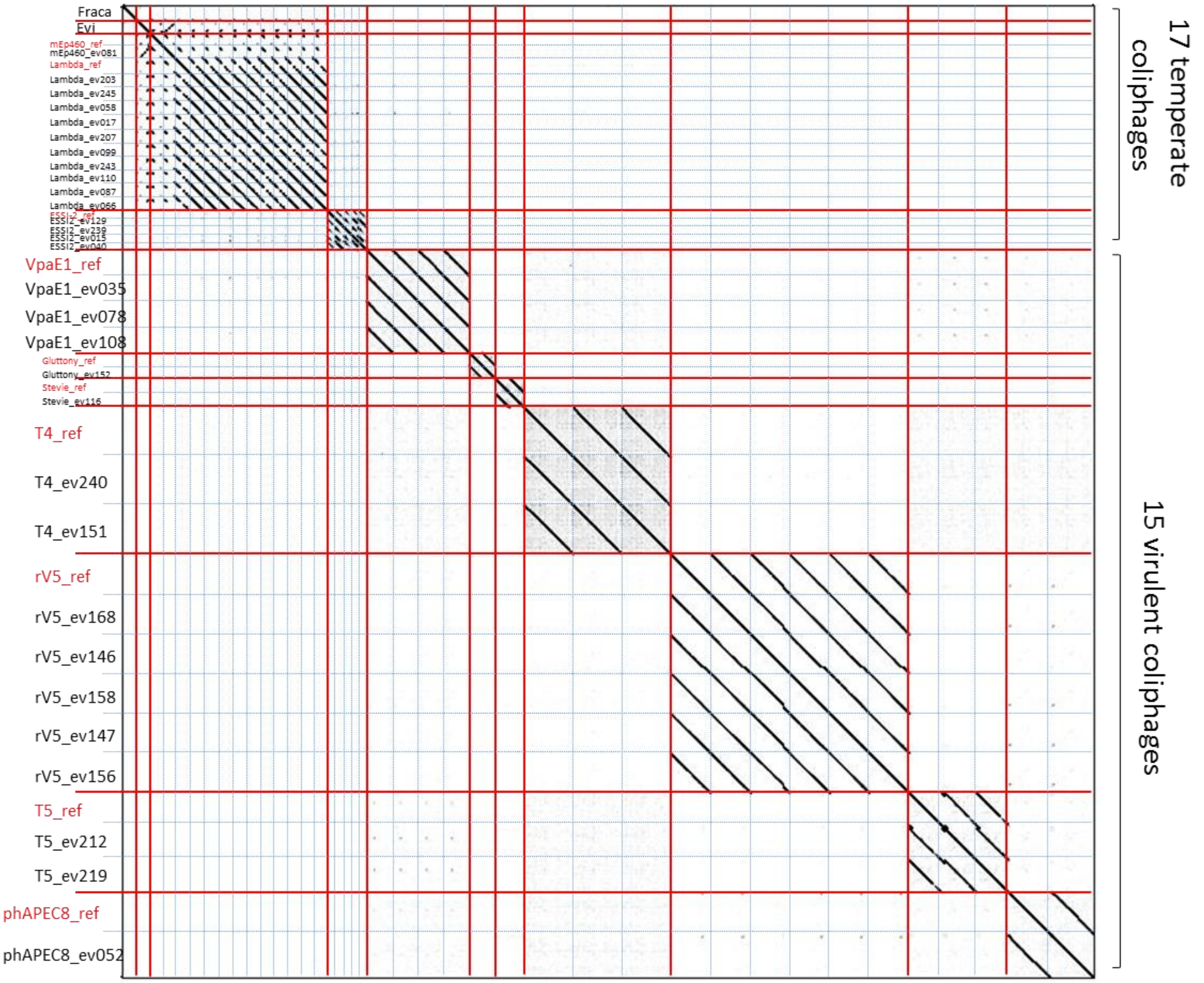

Supplementary Figure 6 : Dot plot analysis of 32 coliphage genomes found in the viromes of 1-year-old infants

Genomes were compared to each other and to reference sequences using Gepard[67] and with a word size of 10. Red lines separate phage clusters (genera) and blue lines separate genomes within the clusters. Intestinal coliphages are in black. Reference phages are in red.

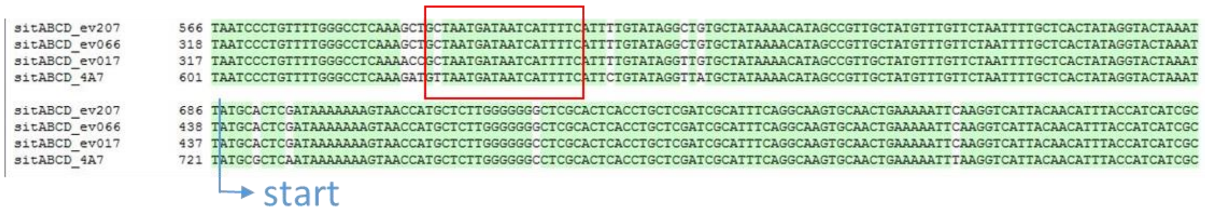

Supplementary Figure 7: A *fur* box is present upstream of the Lambda-encoded *sitABCD* operons. The box is indicated in red on the alignment of the four *sitABCD* upstream regions (one per lambda species).

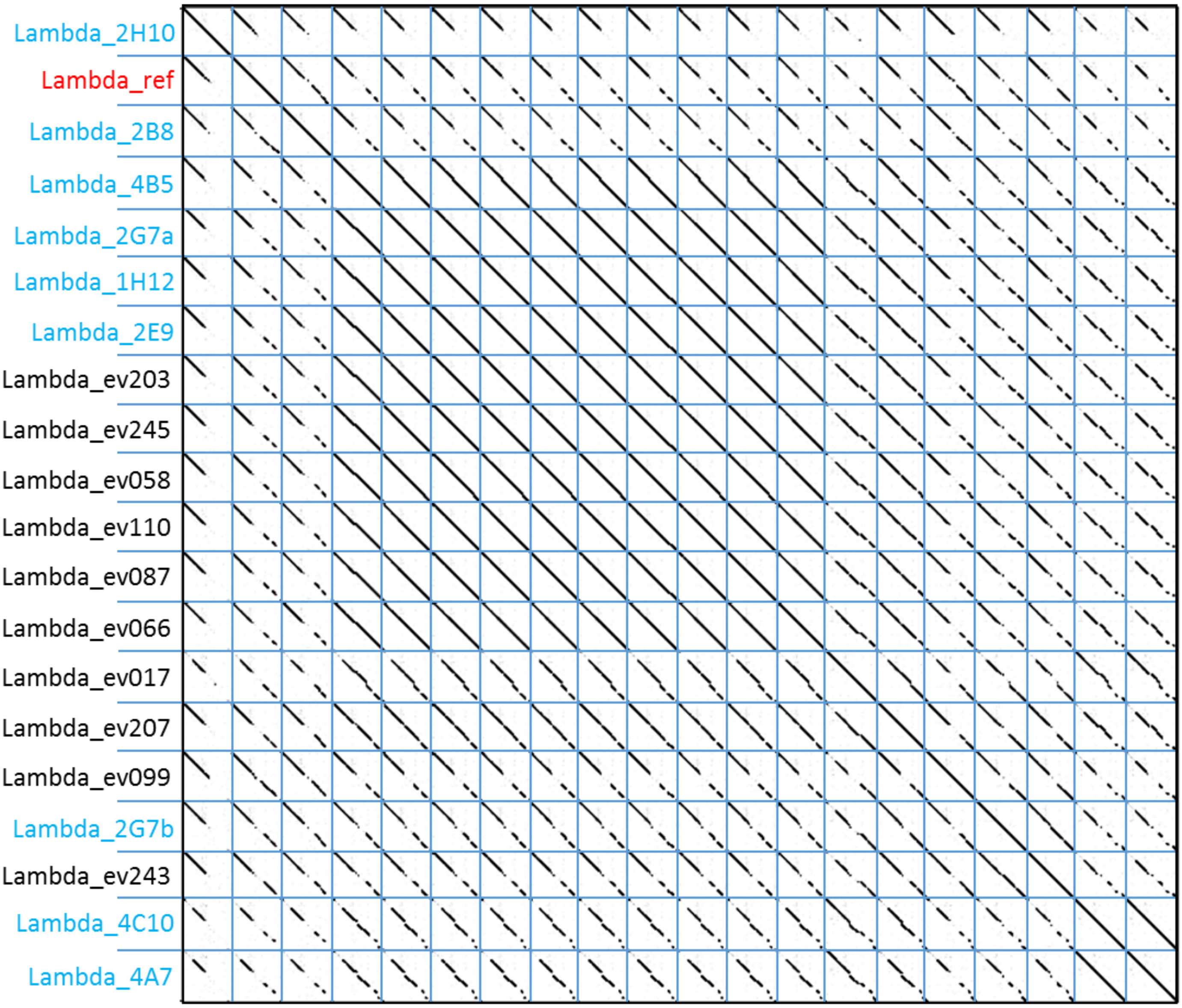

Supplementary Figure 8: Dot plot analysis of 19 lambda-like coliphages genomes. Ten coliphages isolated in the viromes of 1-year-olds (black) and 9 coliphages produced by natural strains isolated at 1 year (blue) are compared with Gepard[67].

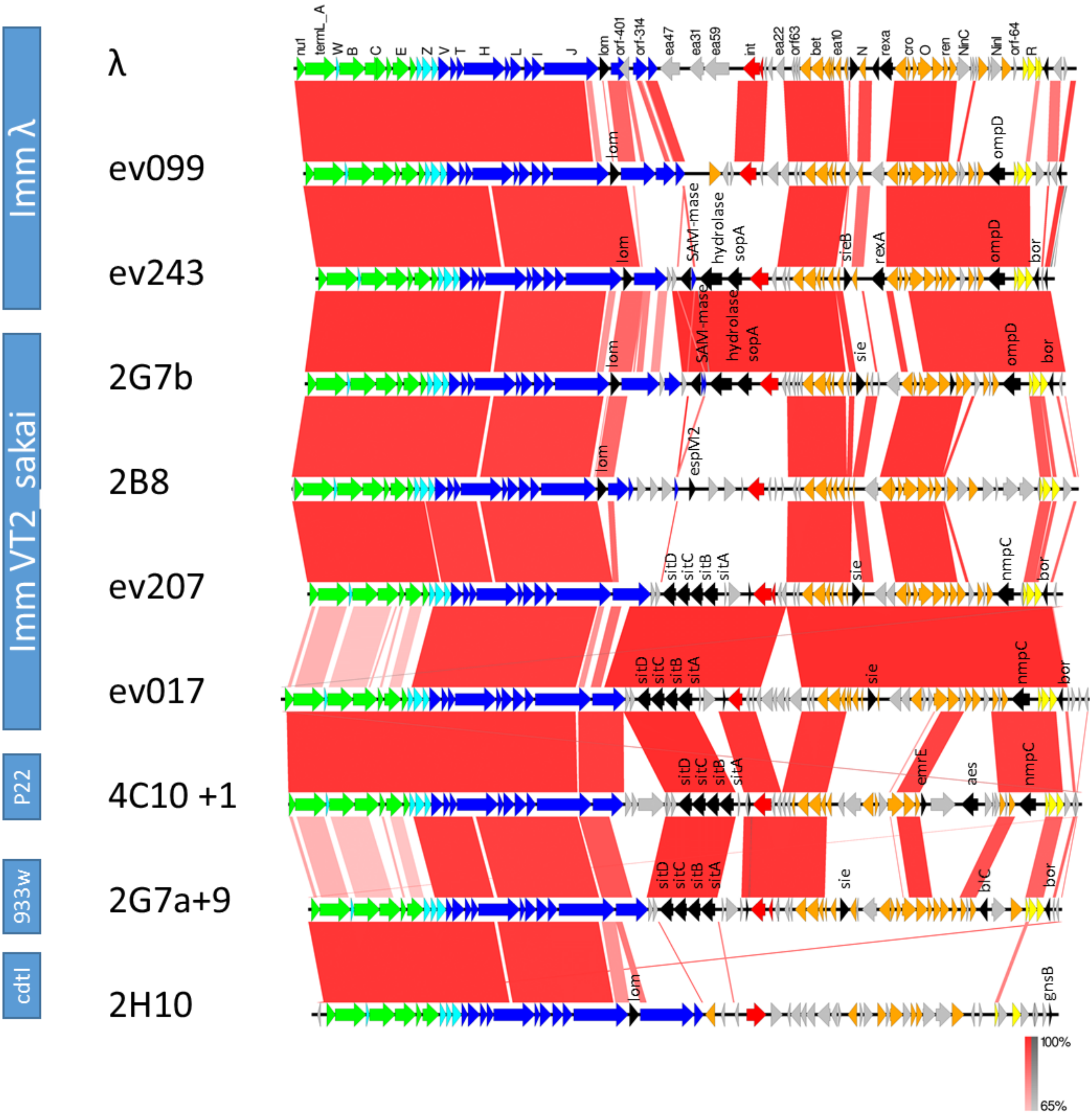

Supplementary Figure 9: Comparisons of lambda genomes. Alignment of reference λ together with representatives of all lambda species isolated from the cohort (genomes were aligned with BLASTn in Easyfig). Genomes are grouped according to their predicted immunity cluster, based on CI similarities (left column). The core (shown as pink to red areas connecting genomes, as function of identity %) and accessory genes (indicated by the white areas) are highlighted. The morons with predicted functions (genes colored in black) are indicated.

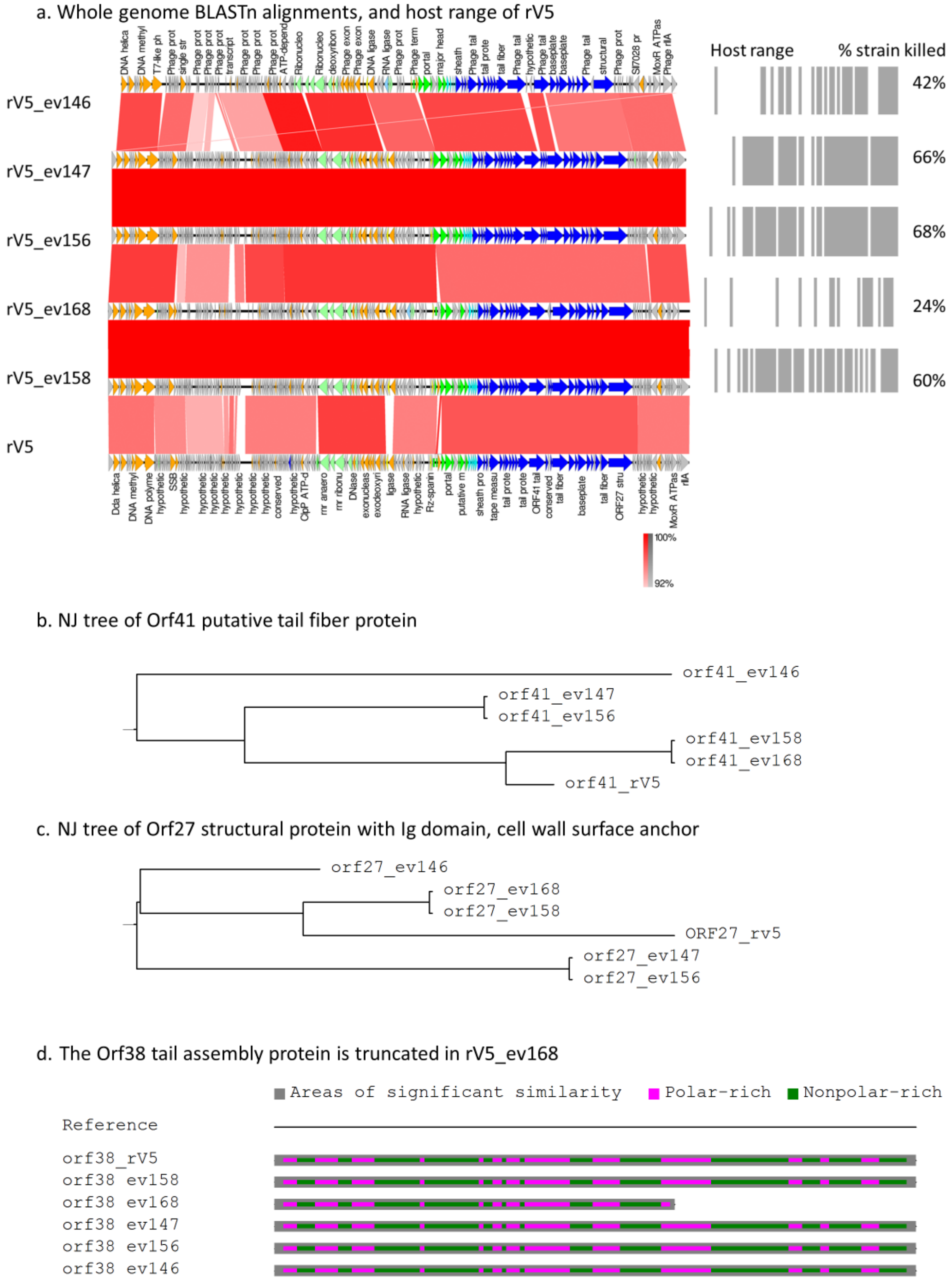

Supplementary Figure 10: the tail proteins Orf27 and Orf41 of rV5 are diverse and cluster together with their respective phage host range. a) Global genome alignments of all rV5, and their host range. Tail proteins are indicated in dark blue. b) Neighbor-Joining tree of Orf41. c) Neighbor-joining tree of Orf27. d) Orf38 protein alignment showing its C-ter truncated version in phage ev168.

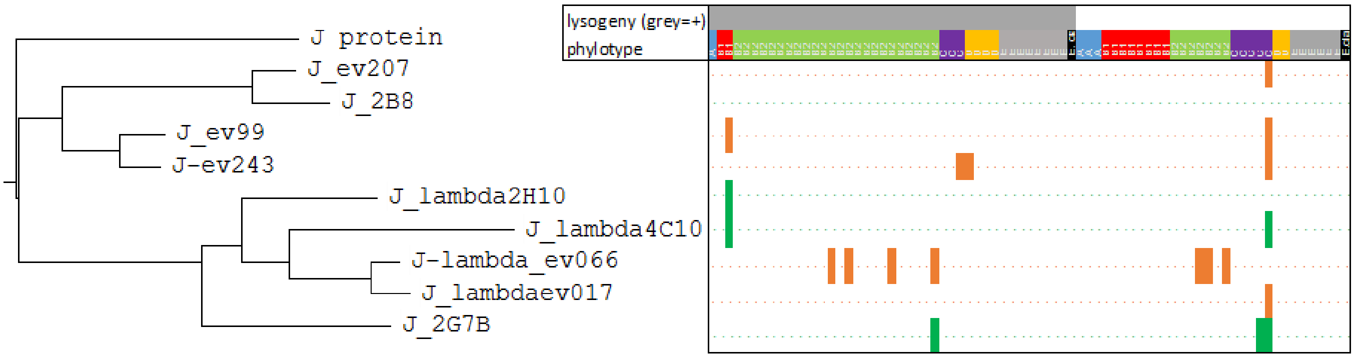

Supplementary Figure 11. The J proteins of lambda phages are diverse, but do not match with their respective phage host ranges. Left: Neighbor-Joining tree of J proteins, right: strains infected by each lambda species (same color code as in Fig. 3). Strains have been reordered as a function of i) their lysogenic character (grey bar above the matrix), ii) their phylotype (blue A, red B1, green B2, purple C, orange D, grey E, black E. clade)

Supplementary Table 1: Percentage of viromes containing infective phages isolated on MAC1403 and C indicator strains.

	Indicator strain		
	MAC1403	C	At least one of the 2
**Fraction of viromes (N=648) containing cultivable coliphages (%)**	14.8	17.3	23.7
