# Supplementary material for: Virulent coliphages in 1-year-old children fecal samples are fewer, but more infectious than temperate coliphages
Source: Nat Commun. 2020 Jan 17;11:378. doi: 10.1038/s41467-019-14042-z (PMC6969025; doi:10.1038/s41467-019-14042-z)
Supplement: Supplementary file 3 — Reporting Summary [file 41467_2019_14042_MOESM3_ESM.pdf]

nature research

Corresponding author(s): Marie-Agnès Petit

Last updated by author(s): november 7th 2019

Reporting Summary

Nature Research wishes to improve the reproducibility of the work that we publish. This form provides structure for consistency and transparency in reporting. For further information on Nature Research policies, see Authors & Referees and the Editorial Policy Checklist.

Statistics

For all statistical analyses, confirm that the following items are present in the figure legend, table legend, main text, or Methods section.

n/a	Confirmed	
☐	☒	The exact sample size (*n*) for each experimental group/condition, given as a discrete number and unit of measurement
☐	☒	A statement on whether measurements were taken from distinct samples or whether the same sample was measured repeatedly
☐	☒	The statistical test(s) used AND whether they are one- or two-sided<br>*Only common tests should be described solely by name; describe more complex techniques in the Methods section.*
☒	☐	A description of all covariates tested
☒	☐	A description of any assumptions or corrections, such as tests of normality and adjustment for multiple comparisons
☐	☒	A full description of the statistical parameters including central tendency (e.g. means) or other basic estimates (e.g. regression coefficient) AND variation (e.g. standard deviation) or associated estimates of uncertainty (e.g. confidence intervals)
☒	☐	For null hypothesis testing, the test statistic (e.g. *F*, *t*, *r*) with confidence intervals, effect sizes, degrees of freedom and *P* value noted<br>*Give P values as exact values whenever suitable.*
☒	☐	For Bayesian analysis, information on the choice of priors and Markov chain Monte Carlo settings
☒	☐	For hierarchical and complex designs, identification of the appropriate level for tests and full reporting of outcomes
☒	☐	Estimates of effect sizes (e.g. Cohen's *d*, Pearson's *r*), indicating how they were calculated

Our web collection on statistics for biologists contains articles on many of the points above.

Software and code

Policy information about availability of computer code

Data collection	*Provide a description of all commercial, open source and custom code used to collect the data in this study, specifying the version used OR state that no software was used.*
Data analysis	*Provide a description of all commercial, open source and custom code used to analyse the data in this study, specifying the version used OR state that no software was used.*

For manuscripts utilizing custom algorithms or software that are central to the research but not yet described in published literature, software must be made available to editors/reviewers. We strongly encourage code deposition in a community repository (e.g. GitHub). See the Nature Research guidelines for submitting code & software for further information.

Data

Policy information about availability of data

All manuscripts must include a data availability statement. This statement should provide the following information, where applicable:

- Accession codes, unique identifiers, or web links for publicly available datasets
- A list of figures that have associated raw data
- A description of any restrictions on data availability

All strains and phages described in the study are available upon request.

Field-specific reporting

Please select the one below that is the best fit for your research. If you are not sure, read the appropriate sections before making your selection.

☐ Life sciences ☐ Behavioural & social sciences ☒ Ecological, evolutionary & environmental sciences

October 2018

Ecological, evolutionary & environmental sciences study design

All studies must disclose on these points even when the disclosure is negative.

Study description	Study of coliphages present in fecal samples of 648 1-year-old children
Research sample	samples were faeces
Sampling strategy	collected by the parents of the child, and sent to the research center
Data collection	data were stored and recorded by the Staten Serum Institute, Copenhagen, Denmark.
Timing and spatial scale	Children were born between march 2009 and march 2011, and feces collected at 1 year of age, except for 58 samples, collected at 2 years. All children live in the Copenhagen area.
Data exclusions	No data were excluded from the analysis
Reproducibility	As stated in the text, all infection experiments (166 phages x 75 strains) were done twice, and if results were discordant, a third test was done.
Randomization	No groups were formed.
Blinding	samples were anonymous.

Did the study involve field work? ☐ Yes ☒ No

Reporting for specific materials, systems and methods

We require information from authors about some types of materials, experimental systems and methods used in many studies. Here, indicate whether each material, system or method listed is relevant to your study. If you are not sure if a list item applies to your research, read the appropriate section before selecting a response.

Materials & experimental systems

n/a	Involved in the study	
☒	☐	Antibodies
☒	☐	Eukaryotic cell lines
☒	☐	Palaeontology
☒	☐	Animals and other organisms
☐	☒	Human research participants
☒	☐	Clinical data

Methods

n/a	Involved in the study	
☒	☐	ChIP-seq
☒	☐	Flow cytometry
☒	☐	MRI-based neuroimaging

Human research participants

Policy information about studies involving human research participants

Population characteristics	Most children were 1 year-old, and the cohort is unselected.
Recruitment	Mothers of participants were volunteers willing to cooperate in the research aiming at understanding asthma. Their child was followed by the COPSAC clinic free of charge during a 6 years period, from birth on.
Ethics oversight	The study was approved by the Ethics Committee of Copenhagen (H-B-2008-093) and the Danish Data Protection Agency (2015-41-3696)

Note that full information on the approval of the study protocol must also be provided in the manuscript.
